# Antibacterial Properties of Nonsteroidal Anti-inflammatory Drugs and Proton Pump Inhibitors Against Enterococcus faecalis: An In Vitro Comparative Study

**DOI:** 10.7759/cureus.85125

**Published:** 2025-05-31

**Authors:** Sufia Parveen, Navin Mishra, Md Jawed Akhtar

**Affiliations:** 1 Department of Conservative Dentistry and Endodontics, Buddha Institute of Dental Sciences and Hospital, Patna, IND; 2 Department of Conservative Dentistry and Endodontics, Post Graduate Institute of Dental Education and Research, Indira Gandhi Institute of Medical Sciences, Patna, IND; 3 Department of Anatomy, Indira Gandhi Institute of Medical Sciences, Patna, IND

**Keywords:** diclofenac sodium, enterococcus faecalis, nsaids, proton pump inhibitor, triple antibiotic paste

## Abstract

Background

*Enterococcus faecalis* is a common cause of persistent endodontic infections due to its resistance to conventional treatments. While triple antibiotic paste (TAP) is effective, concerns about antibiotic resistance have led to interest in nonantibiotic alternatives. Nonsteroidal anti-inflammatory medications (NSAIDs) and proton pump inhibitors (PPIs) have shown promising antibacterial activity. This study aimed to compare the efficacy of diclofenac sodium (DS), TAP, and their combinations with pantoprazole against *E. faecalis* in vitro.

Methodology

This in vitro study evaluated the antibacterial efficacy of DS, TAP, and pantoprazole (a PPI), both individually and in combination, against *E. faecalis* (ATCC 29212). Standardized bacterial suspensions were tested using the agar well diffusion method on Mueller-Hinton agar plates. A total of 140 plates were divided across five groups to assess the antimicrobial zones of inhibition. Test compounds were freshly prepared and introduced into wells under aseptic conditions, followed by incubation at 37°C for 24 hours. Inhibition zones were measured digitally in mm, and data were statistically analyzed using analysis of variance and t-tests, with significance set at p-values <0.05.

Results

In this study, TAP exhibited the highest antibacterial activity among the standard drugs, followed by DS, while PPI showed the least efficacy. However, when combined with PPI, both TAP and DS demonstrated significantly enhanced zones of inhibition against *E. faecalis*, with the TAP + PPI group showing the greatest effect (40.05 ± 0.91 mm), followed by DS + PPI (37.06 ± 0.90 mm). These differences were statistically significant (p < 0.0001), indicating a synergistic benefit from combining PPI with either TAP or DS.

Conclusions

The combination of TAP + PPI showed the highest antibacterial efficacy against *E. faecalis*, followed by DS + PPI, TAP, DS, and PPI alone. Drug combinations demonstrated significantly greater inhibition zones compared to individual agents, indicating enhanced antimicrobial activity.

## Introduction

*Enterococcus faecalis* is a Gram-positive facultative anaerobic bacterium that has become a major problem in endodontic infections because it can remain in root canals even after standard treatments [1]. This bacterium is responsible for a large number of long-lasting periradicular infections. Studies have shown that it is present in 24-77% of cases when root canal procedures did not work [2]. *E. faecalis* is clinically important because it has many virulence features, such as the ability to live as a facultative anaerobe, penetrate dentinal tubules, build biofilms, and resist conventional antimicrobial drugs [3]. Because of these traits, it is very hard to manage the complicated root canal system. This often leads to treatment failures and the need for other treatment methods. Calcium hydroxide (Ca(OH)₂) was the best intracanal medicine for decades as it has an alkaline pH and kills germs. However, there is increasing evidence that it does not work very well against *E. faecalis* biofilms. Studies have shown that up to 40% of those who get standard treatment survive [4]. When compared to triple antibiotic paste (TAP), which is made up of metronidazole, ciprofloxacin, and minocycline, the problems with Ca(OH)₂ were further clarified. Studies have shown that Ca(OH)₂ can reduce bacteria in dentin to a depth of around 200 μm, whereas TAP can go as deep as 400 μm, making it a better antibacterial agent [5,6]. Still, the extensive use of TAP raises serious concerns about antibiotic resistance and possible consequences on the health of stem cells during regenerative endodontic treatments [7]. Researchers are looking at nonantibiotic molecules that might have antibacterial capabilities because antibiotic resistance is becoming a major concern. It is interesting that some groups of medications that were made for other medical objectives, such as antihistamines, antipsychotics, and statins, have been shown to kill bacteria [8]. Nonsteroidal anti-inflammatory medications (NSAIDs) have demonstrated very good effectiveness among these. NSAIDs are mostly used to relieve pain and inflammation, but they also seem to have their own antibacterial effects that work differently than regular antibiotics. Diclofenac sodium (DS) is a popular NSAID that has been shown to kill both Gram-positive and Gram-negative bacteria by stopping DNA synthesis [9]. NSAIDs are especially appealing as possible additions to endodontic therapy as they can perform two things at once.

Proton pump inhibitors (PPIs), which are commonly used to treat acid-peptic diseases, have also shown surprising antibacterial effects. Pantoprazole, a type of PPI, has been proven to work better against endodontic pathogens when used with Ca(OH)₂ [10]. The suggested process involves interfering with bacterial proton pumps, which are important for maintaining the pH level inside cells stable. PPIs could work well with other antimicrobial methods and may even help stop resistance from developing by working differently.

Despite promising findings, regional studies comparing NSAIDs and PPIs against *E. faecalis* remain scarce. This knowledge gap in our region motivated us to investigate these nonantibiotic alternatives. This study aims to evaluate and compare the antibacterial efficacy of DS, TAP, and their combinations with pantoprazole against *E. faecalis* in vitro.

## Materials and methods

We conducted a comprehensive in vitro study to compare the antibacterial properties of three treatment approaches, namely, TAP, DS, and pantoprazole, both individually and in combination.

Material preparation

We obtained pharmaceutical-grade powders of all test compounds (ciprofloxacin, metronidazole, minocycline, DS, and pantoprazole) from Sigma-Aldrich, India. For bacterial culture, we used the standard *E. faecalis* strain ATCC 29212 maintained in brain-heart infusion broth. Before testing, we standardized all bacterial suspensions to a 0.5 McFarland standard (approximately 1.58 × 10⁸ CFU/mL) using turbidity measurements.

Sample size

The sample size (n = 140 plates) was calculated using G*Power 3.1 software [11]. For Step 1 (multiple group comparisons: DS, PPI, TAP), a one-way analysis of variance (ANOVA) was selected with an effect size (f) of 0.8, α = 0.05, and power (1-β) = 0.80, yielding 20 plates per group (total n = 60). For Step 2 (pairwise comparisons: TAP + PPI versus DS + PPI), an independent t-test was used with the same parameters, requiring 40 plates per group (total n = 80). This ensured adequate statistical precision while accounting for intergroup variability [12].

Experimental design

The study consisted of two steps to comprehensively assess both individual and combined drug effects. Step 1: standard drug formulations (baseline groups) (n = 60 plates, 20 plates for each group): Group 1 (DS): prepared by dissolving 100 μg DS powder in 1 mL sterile distilled water to achieve a 100 μg/mL therapeutic concentration, based on prior studies demonstrating efficacy against Gram-positive bacteria [13]; Group 2 (PPI): pantoprazole was dissolved in distilled water to obtain a working concentration of 12.5 μg/m: [12]; and Group 3 (TAP): a standardized 1:1:1 mixture containing equal parts metronidazole (1 mg/mL), ciprofloxacin (1 mg/mL), and minocycline (1 mg/mL) in sterile distilled water (1:1:1 w/v) [12].

Step 2: experimental drug combinations with PPI (n = 80 plates, 40 plates for each group): Group 4 (TAP + PPI): combined in equal volumes (1:1 ratio) immediately before testing to minimize compound degradation while evaluating potential synergistic enhancement of TAP’s antibacterial activity; and Group 5 (DS + PPI): similarly mixed in a 1:1 proportion to assess whether this completely nonantibiotic combination could provide clinically relevant antimicrobial effects, particularly valuable for patients with antibiotic contraindications.

Agar well diffusion assay

The study employed a standardized agar well diffusion method to evaluate antibacterial efficacy, utilizing Mueller-Hinton agar plates (Merck, Germany) prepared under strict aseptic conditions. Plates were included in the analysis only if they met predefined technical criteria, including uniform agar depth, standardized inoculum (0.5 McFarland), properly formed wells, and absence of contamination or procedural error. Plates not meeting these criteria were excluded to ensure the consistency and validity of the results. For Step 1, testing individual compounds (DS, PPI, and TAP), a total of 60 plates were prepared, with 20 plates allocated to each treatment group to ensure adequate sample size. Step 2, which examined combination therapies (TAP + PPI and DS + PPI), required 80 Mueller-Hinton plates, with 40 plates per combination group, to maintain statistical power of 80% and 95% confidence intervals while accounting for potential biological variability.

The assay protocol followed a carefully standardized procedure beginning with plate preparation, where media was poured to a uniform 4 mm depth for consistency across all tests. Using a flame-sterilized 5 mm diameter cork borer, we created precisely measured wells (5 mm diameter × 2 mm depth) in each plate, maintaining careful spacing between wells to prevent any overlap of inhibition zones. Each plate then received a standardized inoculum of *E. faecalis* adjusted to 0.5 McFarland standard, which was spread evenly across the surface using sterile cotton swabs applied in three perpendicular directions to ensure complete and uniform bacterial coverage.

Test solutions were applied with precision using calibrated micropipettes to deliver exactly 30 μL of each treatment into designated wells, with particular attention paid to avoiding any spillage or overflow that might compromise results. Following preparation, all plates underwent controlled aerobic incubation in a calibrated Thermo Scientific incubator maintained at a constant 37°C (±0.5°C) for exactly 24 hours, with humidity controls in place to prevent agar dehydration during the incubation period. This comprehensive methodology was designed to provide highly reproducible conditions for accurate comparison of antibacterial effects across all test groups while adhering to the highest standards of experimental microbiology.

Outcome measurement

Following incubation, an independent examiner blinded to treatment groups measured inhibition zones using a digital caliper (Mitutoyo, ±0.01 mm accuracy). Three measurements were taken per well at 120° intervals, with the mean value recorded as the zone of inhibition. All measurements were performed under consistent lighting conditions using a standardized viewing box to ensure measurement reliability.

Statistical analysis

All study data were systematically compiled using Microsoft Excel (Office 2019) before being imported into GraphPad Prism version 8.4.3 for comprehensive statistical evaluation. For Step 1, comparing individual compounds (DS, PPI, and TAP), we performed one-way ANOVA with post-hoc Tukey’s test to assess differences in mean inhibition zone diameters across the three treatment groups. Step 2 data, evaluating the combination therapies (TAP + PPI versus DS + PPI), were analyzed using independent samples t-tests. In all analyses, we established statistical significance at p-values <0.05, with results reported as mean ± standard deviation to indicate measurement variability.

## Results

**Figure 1:**
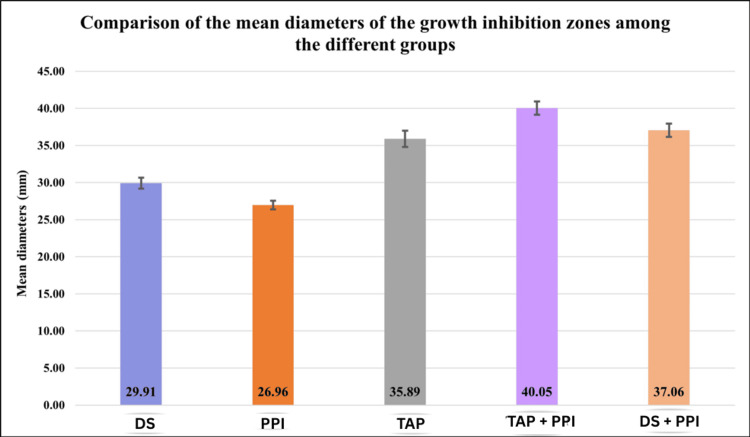
Mean diameters of growth inhibition zones (in mm) among the different groups. DS: diclofenac sodium; PPI: proton pump inhibitor; TAP: triple antibiotic paste

In this present in vitro study, we observed that among the standard drug formulations, TAP exhibited the highest mean zone of inhibition (35.89 ± 1.08 mm), followed by DS, with a mean diameter of 29.91 ± 0.74 mm, and PPI, which showed the lowest activity with a mean of 26.96 ± 0.58 mm. The addition of PPI to TAP resulted in a further enhancement of antibacterial activity, as evidenced by a significantly increased mean inhibition zone of 40.05 ± 0.91 mm in the TAP + PPI group. Similarly, the combination of DS with PPI (DS + PPI) also demonstrated improved efficacy compared to DS alone, with a mean inhibition zone of 37.06 ± 0.90 mm. These differences were statistically significant, with a p-value <0.0001, indicating that the combination of PPI with either TAP or DS resulted in a synergistic or additive enhancement of antibacterial activity against *E. faecalis* (Table 1, Figure 1).

**Table 1:** Comparison of the diameters of the growth inhibition zones among the different groups.

Group number	Drug used	Number of samples (n)	Mean diameter of growth inhibition zones (in mm) (mean ± SD)	P-value
Standard drug formulations				
1	Diclofenac sodium	20	29.91 ± 0.74	<0.0001
2	Proton pump inhibitor	20	26.96 ± 0.58	
3	Triple antibiotic paste	20	35.89 ± 1.08	
Drug combinations				
4	Triple antibiotic paste + proton pump inhibitor	40	40.05 ± 0.91	<0.0001
5	Diclofenac sodium + proton pump inhibitor	40	37.06 ± 0.90	

The intergroup comparison of mean scores was conducted using a post-hoc Tukey test, which revealed statistically significant differences in the mean diameters of growth inhibition zones across all tested groups (p < 0.0001) (Table 2). DS demonstrated significantly greater antibacterial activity than PPI, but significantly less than TAP, TAP combined with PPI, and DS combined with PPI. PPI alone was the least effective, showing significantly smaller zones of inhibition compared to all other groups. TAP showed superior efficacy over DS and PPI but was outperformed by both TAP + PPI and DS + PPI combinations. Among all, the TAP + PPI group exhibited the largest inhibition zones, followed closely by DS + PPI, with the difference between them also being statistically significant. These findings suggest a clear enhancement of antibacterial activity when PPI is combined with either TAP or DS.

**Table 2:** Intergroup comparison of mean diameters of the growth inhibition zones among the different groups.

Group A	Group B	Mean difference	P-value
Diclofenac sodium	Proton pump inhibitor	2.951	<0.0001
	Triple antibiotic paste	-5.984	<0.0001
	Triple antibiotic paste + proton pump inhibitor	-10.14	<0.0001
	Diclofenac sodium + proton pump inhibitor	-7.153	<0.0001
Proton pump inhibitor	Triple antibiotic paste	-8.935	<0.0001
	Triple antibiotic paste + proton pump inhibitor	-13.09	<0.0001
	Diclofenac sodium + proton pump inhibitor	-10.10	<0.0001
Triple antibiotic paste	Triple antibiotic paste + proton pump inhibitor	-4.156	<0.0001
	Diclofenac sodium + proton pump inhibitor	-1.168	<0.0001
Triple antibiotic paste + proton pump inhibitor	Diclofenac sodium + proton pump inhibitor	2.987	<0.0001

## Discussion

The rise of antibiotic resistance has necessitated the exploration of alternative approaches for disinfecting root canals, particularly those involving PPIs and NSAIDs. This study evaluated the antimicrobial potential of NSAIDs and PPIs in comparison to TAP to determine their effectiveness as intracanal medicaments against *E. faecalis*. The bacteria *E. faecalis* were selected based on the foundational work of Haapasalo and Orstavik and continue to be a major etiological factor in persistent endodontic infections due to their capacity to endure hostile environments, penetrate dentinal tubules, and form robust biofilms that are resistant to standard disinfectants and antibiotics [14]. The organism’s biofilm-forming ability, stress tolerance, and genetic adaptability make it a formidable challenge in root canal therapy. These factors make *E. faecalis* the ideal organism for evaluating the efficacy of novel intracanal antimicrobial agents. The agar diffusion method, a classical technique for screening antimicrobial efficacy, remains a cornerstone in microbiological evaluation. Despite certain limitations, such as the dependency on the diffusibility of the medicament in agar, it allows for consistent and comparative analysis of inhibition zones, which is crucial for preliminary screening [15].

TAP, composed of metronidazole, minocycline, and ciprofloxacin, served as the positive control due to its established efficacy against *E. faecalis *[16]. The synergistic activity of its components contributes to its potent antibacterial effect. Minocycline inhibits protein synthesis at the ribosomal level, metronidazole disrupts DNA structure in anaerobic environments, and ciprofloxacin targets bacterial DNA gyrase [17]. Moreover, their collective anti-inflammatory and pro-regenerative properties enhance their value in endodontic therapy. However, concerns remain regarding cytotoxicity and the development of resistance, particularly with repeated use of antibiotic combinations in regenerative endodontic protocols.

Among NSAIDs, DS has shown notable antibacterial activity due to mechanisms such as inhibition of DNA synthesis, interference with bacterial membrane potential, and downregulation of efflux pumps [18,19]. This multi-targeted antimicrobial behavior was validated in a study by Chockattu et al., where DS demonstrated significant activity against *E. faecalis* [20]. Pantoprazole, a commonly prescribed PPI, emerged as a novel antimicrobial agent by virtue of its ability to disrupt proton gradients in bacterial cells. Its interference with intracellular pH regulation leads to membrane destabilization and impaired metabolic function in bacteria [21]. While its standalone antibacterial effect was modest in this study, its role became more prominent when used in combination with either DS or TAP.

Our comparative analysis revealed that TAP demonstrated the largest zone of inhibition among the individual agents, followed by DS and PPI. These results are in agreement with prior research by Prabhakar et al., who highlighted the rapid bactericidal effect of TAP in infected root canal systems [22]. Similarly, studies by Valverde et al. and Zancan et al. confirmed the strong activity of TAP against *E. faecalis* biofilms [23,24]. Interestingly, combination therapy markedly enhanced antimicrobial efficacy. The TAP + PPI group exhibited the highest mean zone of inhibition (40.05 ± 0.91 mm), followed by DS + PPI (37.06 ± 0.90 mm), with statistically significant differences compared to individual agents. This synergism is likely due to the complementary mechanisms of action. While TAP eradicates bacteria via direct antimicrobial pathways, the addition of PPI likely augments drug penetration or disrupts microbial pH homeostasis, weakening the biofilm matrix and improving susceptibility to antibiotics. Tilokani et al. observed a similar trend, where the TAP + PPI and DS + PPI combinations outperformed the individual agents, highlighting the additive or synergistic potential of combining PPIs with other drugs [12]. These findings were further substantiated by Deepak and Antony, who demonstrated that TAP + PPI combinations are effective even against resistant *E. faecalis* strains [25]. From a clinical standpoint, these results offer promising implications for intracanal medicament development. The enhanced activity of TAP and DS when combined with pantoprazole suggests that nonantibiotic adjuvants such as PPIs can improve the efficacy of existing antimicrobial regimens. This is particularly important in the context of antibiotic stewardship, where the goal is to minimize antibiotic use without compromising treatment outcomes. The combination of DS + PPI is especially noteworthy because it offers a nonantibiotic alternative with substantial antimicrobial efficacy. This could be especially beneficial in patients with contraindications to antibiotics or in cases where antibiotic resistance is a concern. Furthermore, the potential dual benefit of NSAIDs in reducing inflammation and microbial load may enhance periapical healing and overall treatment success. Even though in vitro findings are promising, further in vivo and clinical studies are needed to assess pharmacokinetics, tissue interactions, and safety profiles to validate clinical applicability.

Study limitations

This study has a few limitations. In this in vitro study, we only used a single bacterial strain (*E. faecalis*), without a biofilm model, did not include cytotoxicity testing on human tissues, had a short observation period, and did not confirm the exact mechanism behind the observed drug synergy.

## Conclusions

Based on the findings of this study, the combination of TAP with a PPI (TAP + PPI) demonstrated the highest antibacterial activity against *E. faecalis*, followed by the combination of DS with PPI (DS + PPI), then TAP alone, DS alone, and finally PPI alone. These results suggest that combining agents, particularly those with complementary mechanisms, can significantly enhance antimicrobial efficacy in endodontic infections. Utilizing such combinations, especially those involving nonantibiotic agents, may offer a promising strategy in clinical practice by reducing the risk of systemic side effects and minimizing the potential for developing antibiotic resistance.
